# Say it right: measuring the impact of different communication strategies on the decision to get vaccinated

**DOI:** 10.1186/s12889-023-16047-2

**Published:** 2023-06-16

**Authors:** Vivian I. Avelino-Silva, Sofia Natalia Ferreira-Silva, Maria Eduarda Muniz Soares, Ricardo Vasconcelos, Luiz Fujita, Tainah Medeiros, Carolina Luisa Alves Barbieri, Marcia Thereza Couto

**Affiliations:** 1 Department of Infectious and Parasitic Diseases, Faculdade de Medicina FMUSP, Universidade de Sao Paulo, Av. Dr. Arnaldo, 455, Cerqueira Cesar, Sao Paulo – SP, 01246-903 Brazil; 2 Departamento de Medicina Preventiva, Faculdade de Medicina FMUSP, Universidade de Sao Paulo, Av. Dr. Arnaldo, 455, Cerqueira Cesar, Sao Paulo – SP, 01246-903 Brazil; 3 Faculdade Israelita de Ciências da Saúde Albert Einstein, Hospital Israelita Albert Einstein, Rua Comendador Elias Jafet, 755, Morumbi, Sao Paulo – SP, 05653-000 Brazil; 4 Centro de Pesquisas Clínicas, Hospital das Clínicas, Faculdade de Medicina FMUSP, Universidade de Sao Paulo, R. Dr. Ovídio Pires de Campos, 225, Cerqueira Cesar, Sao Paulo – SP, 05403-010 Brazil; 5 Baioque, R. Augusta, 101 - Sala 109 - Consolacao, Sao Paulo – SP, 01305-000 Brazil; 6 Instituto Butantan, Av. Vital Brasil, 1500, Butanta, Sao Paulo – SP, 05503-900 Brazil

**Keywords:** Vaccines, Vaccination hesitancy, Surveys and questionnaires, Random allocation, Health communication

## Abstract

**Background:**

Vaccine hesitancy is a concerning menace to the control of vaccine-preventable diseases. Effective health communication could promote an overall understanding of the importance, risks, and benefits of vaccination and reduce vaccine hesitancy.

**Methods:**

In this survey, four fictitious newspaper articles addressing an emerging bogus disease and its vaccine were randomly assigned to participants. The first version focused on information about the disease; the second was akin to the first, including a case description and image. The third version focused on vaccine safety/efficacy; the fourth version was like the third, including a case description and image. After reading a single version of the article, participants responded if they would take the vaccine and if they would vaccinate their children. We used chi-squared tests for comparisons and investigated interactions with vaccine-hesitant attitudes.

**Results:**

We included 5233 participants between August/2021 and January/2022; 790 were caregivers of a child ≤ 5 years old, and 15% had prior vaccine hesitancy. Although most declared intention to take the vaccine, the percentage was highest among those exposed to the newspaper article focusing on the vaccine safety/efficacy with the case description and picture (91%; 95% confidence interval 89–92%), and lowest among participants exposed to the article focusing on the disease with no case description (84%; 95% confidence interval 82–86%). Similar trends were observed in the intention of offspring vaccination. We found evidence of effect modification by vaccine-hesitant attitudes, with a higher impact of communication focusing on vaccine safety/efficacy compared to that focusing on disease characteristics among hesitant participants.

**Conclusion:**

Communication strategies focusing on different aspects of the disease-vaccine duet may impact vaccine hesitancy, and storytelling/emotive imagery descriptions may improve risk perception and vaccine uptake. Moreover, the effect of message framing strategies may differ according to previous vaccine hesitant attitudes.

## Introduction

Mass vaccination has been adopted for almost two centuries to prevent infectious diseases such as smallpox, polio, tetanus, pertussis, hepatitis A and B, HPV, and yellow fever [1]. Moreover, mass vaccination can prevent clinical and financial repercussions associated with those diseases and has been consistently associated with major public health developments [2].

Vaccine effectiveness depends not only on the availability of resources but also on mass acceptance and uptake of accessible vaccines. Vaccine hesitancy is defined as the delay in acceptance or refusal to vaccinate despite the availability of vaccination services [3]. Studies conducted in affluent and resource-limited settings support vaccine hesitancy as a central driver of vaccine noncompliance [4, 5]. In recent studies, vaccine hesitancy has been associated with a reduction in vaccine coverage and outbreaks of vaccine-preventable diseases in several regions [6–8]. More recently, during the COVID-19 pandemic, political ideologies have emerged as additional influencing factors for vaccine hesitancy. For instance, former right-wing presidents of Brazil and the USA have issued declarations opposing COVID-19 vaccines, and studies from both countries demonstrated that political alignment has been a strong driver of hesitancy towards COVID-19 vaccination [9, 10].

Health communication strategies are essential tools to address vaccine hesitancy [11–13]. However, few studies with inconsistent results have explored evidence-based strategies to improve communication and reduce vaccine hesitancy [14]. Interestingly, communication strategies that positively affect specific populations may have a null or even detrimental effect in other subgroups, notably based on prior hesitant attitudes or cultural specificities [15, 16].

In this randomized experiment, we investigate the effect of different communication strategies on the intention to receive a vaccine for a bogus emerging viral disease and the intention of offspring vaccination. We also explored interactions between communications strategies and prior vaccine-hesitant attitudes.

## Methods

**Fig. 1:**
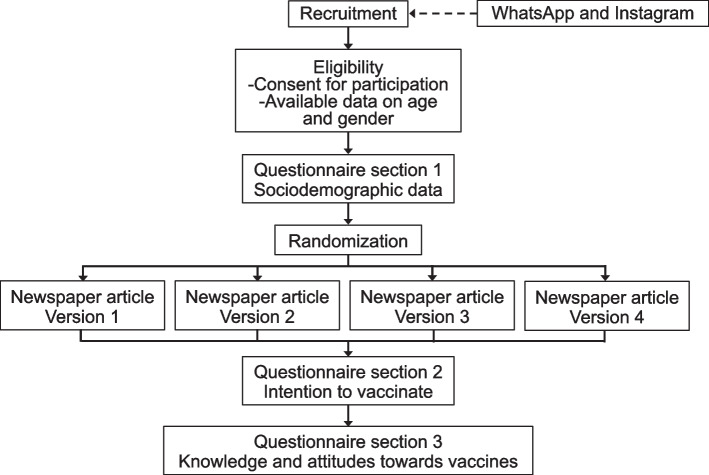
Schematic of study recruitment and procedures

In this survey experiment, we recruited participants ≥ 18 years old living in Brazil using social media appliances (Instagram and WhatsApp) disseminated by the study investigators, the study's official social media profiles, and the official profiles of Faculdade de Medicina da Universidade de Sao Paulo. Participants responded to a self-administered electronic questionnaire including three sections: 1-demographics; 2-intention to receive a vaccine to prevent an emerging bogus disease and to vaccinate their offspring if applicable; and 3-knowledge/attitudes regarding vaccines. Before section 2, each participant was exposed to a fictitious newspaper article describing a bogus disease and its vaccine. Four versions of the newspaper article were randomly assigned to study participants, with each participant having access to a single version (Fig. 1). The articles were designed to address if different message-framing strategies [14] could influence participants' intentions to receive the vaccine and to vaccinate their offspring. The first version focused on information about the disease's clinical and epidemiological characteristics and outcomes (fear-based messaging); the second version was akin to the first version but incorporated a description and picture of a child affected by the disease (storytelling/emotive imagery). The third version focused on data concerning the vaccine, with more detailed information on vaccine safety and efficacy (science-based messaging); the fourth version was similar to the third article but incorporated a description and picture of a child affected by the disease, as presented in Table 1. The study investigators created the newspaper articles ad-hoc for this study with the support of professional health journalists. Participants allocated to versions 1 to 4 of the newspaper article are hereafter referred to by the corresponding groups 1 to 4. After reading the article, participants were asked if they would take the vaccine; parents or legal guardians of a child ≤ 5 years old were also asked if they would vaccinate their children. Responses were collected using close-ended, single-choice answers (yes; no; I don't know; I don't want to declare).

**Table 1:** Newspaper articles describing an emerging bogus disease and its vaccine

Newspaper article Version 1 Group 1	The Human Acute Meningitis Virus (HAMV) epidemic, which causes severe meningitis and has already resulted in more than 500 deaths across five continents since February 2020, has reached yet another country. New Zealand, which had remained free of cases of the disease for eight months, has recorded its first infected patient. The virus is transmitted by airborne droplets—like the flu—and causes severe headaches, fever, vomiting, and convulsions. The disease primarily affects children between the ages of two and eight, and the case-fatality rate is approximately 50% in this age group. Among survivors, cases of sequelae include epilepsy and intellectual disability. So far, there is no specific treatment against the disease, but a vaccine against the virus is in development. It is expected to be available within a few weeks in Brazil, with distribution led by the national health system
Newspaper article Version 2 Group 2	The Human Acute Meningitis Virus (HAMV) epidemic, which causes severe meningitis, has resulted in more than 500 deaths across five continents since February 2020. The virus is transmitted by airborne droplets—like the flu—and causes severe headaches, fever, vomiting, and convulsions. The disease primarily affects children between the ages of two and eight, and the case-fatality rate is approximately 50% in this age group. Among survivors, cases of sequelae include epilepsy and intellectual disability

	In Brazil, four-year-old Pedro was the first case observed in the country, generating much commotion. The child was hospitalized for 28 days at Hospital das Clinicas, Universidade de Sao Paulo, and survived. Still, the infection resulted in irreversible hearing loss There is still no specific treatment for HAMV, but a vaccine against the virus is under development. Pedro's father, mechanic Valdir Amilton, 39, regretted that the immunization was not available sooner: "Now we will have to cope with this disability, but at least other people will not have to go through it." The vaccine is expected to be available within a few weeks in Brazil, with distribution led by the national health system
Newspaper article Version 3 Group 3	Several countries, including Brazil, have already implemented vaccination campaigns against the Human Acute Meningitis Virus (HAMV), which causes severe meningitis and has resulted in more than 500 deaths across five continents since February 2020. The vaccine is given as a single dose and was found to be safe in two large studies with more than 10,000 people in the United Kingdom and Australia. Mild adverse reactions were observed in only 10% to 15% of study participants. Adverse events include pain at the injection site and low-grade fever. According to experts and health authorities, the vaccine is highly effective, protecting more than 95% of children ten days after vaccination. In Brazil, the vaccine is available at public primary care clinics, private clinics, and select drugstores
Newspaper article Version 4 Group 4	Several countries, including Brazil, have already implemented vaccination campaigns against the Human Acute Meningitis Virus (HAMV), which causes severe meningitis and has resulted in more than 500 deaths across five continents since February 2020 The vaccine is given as a single dose and was found to be safe in two large studies with more than 10,000 people in the United Kingdom and Australia. Mild adverse reactions were observed in only 10% to 15% of study participants. Adverse events include pain at the injection site and low-grade fever. According to experts and health authorities, the vaccine is highly effective, protecting more than 95% of children ten days after vaccination

	Four-year-old Pedro was the first case observed in Brazil, generating much commotion. The child was hospitalized for 28 days at Hospital das Clinicas, Universidade de Sao Paulo, and survived. Still, the infection resulted in irreversible hearing loss There is still no specific treatment for HAMV, but a vaccine against the virus is under development. Pedro's father, mechanic Valdir Amilton, 39, regretted that the immunization was not available sooner: "Now we will have to cope with this disability, but at least other people will not have to go through it." In Brazil, the vaccine is available at public primary care clinics, private clinics, and select drugstores

The primary dependent variables in our analyses were the reported intention to take the vaccine and the intention of offspring vaccination.

As other studies have shown that the effect of specific message framing strategies may differ according to prior (hesitant) beliefs regarding vaccines [15], we selected six statements that evaluated vaccine hesitant attitudes from part 3 of the questionnaire. We categorized participants who responded "agree" to any of these statements as having prior vaccine hesitancy. The statements used to define vaccine hesitancy were: 1. Healthy children don't need so many vaccines in their first year of life; 2. People with a healthy lifestyle who can care for themselves don't need so many vaccines; 3. It's preferable to gain immunity from the disease than from the vaccine; 4. Very often, it's preferable to face the risk of having the disease than the risk of a vaccine's side effects; 5. Vaccines are needed only when an outbreak or epidemic is ongoing; and 6. An excess of vaccines can be bad for your health.

We used the REDCap platform [17] to develop the electronic survey and collect questionnaire responses.

The DEBRA study has been reviewed and approved by the Ethics Committees at Hospital Israelita Albert Einstein (Nº 5,246,486) and Hospital das Clínicas da Faculdade de Medicina da Universidade de São Paulo (Nº 4,737,962). All subjects or their legal guardian(s) provided informed consent before inclusion in the study. We collected no identifiable private information from study participants. All methods were carried out following relevant guidelines and regulations. Images presented in Table 1 are not of study participants and were taken from Depositphotos™.

We compared demographic characteristics in each group using Kruskal–Wallis tests and chi-squared tests as appropriate, with a 0.05 significance level. We describe counts, percentages, and 95% confidence intervals (CI) for responses concerning the intention to receive a vaccine and to vaccinate offspring in each study group; two by two comparisons were also performed using chi-squared tests. We calculated prevalence ratios comparing groups according to prior hesitant beliefs and explored the presence of multiplicative interaction using Mantel–Haenszel's homogeneity tests. We handled missing as missing completely at random. We used Stata (StataCorp. College Station, TX: StataCorp LP) version 15.1 in all analyses.

## Results

### Demographic characteristics of study participants and vaccine hesitant beliefs

Between August/2021 and January/2022, 6769 individuals provided consent for participation, of whom 5233 informed essential demographic data (age and gender) and were included in the analysis. Table 2 describes the demographic characteristics of study participants, overall and according to group allocation. Groups 1 to 4 comprised 1320, 1337, 1259, and 1317 participants. The median age was 42 years old (interquartile range [IQR] 32–56 years old); most participants were females (68%), and most were white/Caucasians (79%) with university-level education (81%). Participants described a wide variability of religious practices, with a higher percentage of Catholics (38%) and atheists/agnostics or participants reporting no religion (30%). Although the study included participants from all Brazilian federal units, most (67%) were from Sao Paulo State. From the total sample, 790 (15%) were parents or caregivers of a child ≤ 5 years old. Based on responses to questionnaire part 3, we categorized 696 participants (15%) as having prior vaccine hesitancy. We found no statistically significant differences among groups regarding sociodemographic characteristics or prior vaccine hesitancy (Table 2).

**Table 2:** Demographic characteristics and prior vaccine hesitancy of study participants, overall and according to group allocation

	Total *N* = 5233	Group 1 *N* = 1320	Group 2 *N* = 1337	Group 3 *N* = 1259	Group 4 *N* = 1317	*p*-value
Gender (%)^a^						0.237
Male	1632 (31)	428 (33)	395 (30)	375 (30)	434 (33)	
Female	3539 (68)	870 (66)	926 (70)	875 (70)	868 (66)	
Other	24 (< 1)	8 (1)	6 (< 1)	5 (< 1)	5 (< 1)	
Don't want to declare	18 (< 1)	7 (1)	4 (< 1)	1 (< 1)	6 (< 1)	
Median age (IQR)^b^	42 (32–56)	42 (32–56)	41 (30–55)	42 (32–56)	43 (32–57)	0.061
Education (%)^c^						0.834
< Elementary	11 (< 1)	2 (< 1)	4 (< 1)	2 (< 1)	3 (< 1)	
Elementary	10 (< 1)	5 (< 1)	4 (< 1)	0	1 (< 1)	
Middle	18 (< 1)	4 (< 1)	5 (< 1)	4 (< 1)	5 (< 1)	
High	933 (18)	244 (19)	244 (18)	222 (18)	223 (17)	
University	2461 (47)	613 (47)	636 (48)	589 (47)	623 (48)	
Post-graduate	1770 (34)	445 (34)	437 (33)	433 (35)	455 (35)	
Race/skin color (%)^d^						0.847
White/Caucasian	4131 (79)	1026 (78)	1053 (79)	1001 (80)	1051 (80)	
Black	215 (4)	65 (5)	58 (4)	41 (3)	51 (4)	
Mixed	612 (12)	152 (12)	155 (12)	152 (12)	153 (12)	
Asian	198 (4)	53 (4)	55 (4)	42 (3)	48 (4)	
Native	11 (< 1)	4 (< 1)	3 (< 1)	2 (< 1)	2 (< 1)	
Other	16 (< 1)	6 (< 1)	4 (< 1)	4 (< 1)	2 (< 1)	
Don't want to declare	42 (1)	11 (1)	8 (1)	14 (1)	9 (1)	
Religion (%)^e^						0.731
Catholic	1983 (38)	507 (39)	490 (37)	474 (38)	512 (39)	
Protestant	404 (8)	104 (8)	93 (7)	97 (8)	110 (8)	
Kardecist	684 (13)	163 (12)	172 (13)	177 (14)	172 (13)	
Atheist/Agnostic/no religion	1589 (30)	395 (30)	426 (32)	386 (31)	382 (29)	
Jewish	64 (1)	19 (1)	14 (1)	16 (1)	15 (1)	
African-based religions	127 (2)	31 (2)	37 (3)	32 (3)	27 (2)	
Other	161 (3)	49 (4)	47 (4)	28 (2)	37 (3)	
Don't want to declare	215 (4)	48 (4)	57 (4)	48 (4)	62 (5)	
State of residency (%)^f^						0.723
Acre	3 (< 1)	0	1 (> 1)	1 (< 1)	1 (< 1)	
Alagoas	51 (1)	9 (1)	12 (1)	17 (1)	13 (< 1)	
Amapá	4 (< 1)	1 (< 1)	0	1 (< 1)	2 (< 1)	
Amazonas	8 (< 1)	3 (< 1)	2 (< 1)	2 (< 1)	1 (< 1)	
Bahia	115 (2)	25 (2)	27 (2)	29 (2)	34 (3)	
Ceará	38 (1)	16 (1)	5 (< 1)	11 (1)	6 (< 1)	
Distrito Federal	108 (2)	30 (2)	30 (2)	18 (1)	30 (2)	
Espírito Santo	37 (1)	13 (1)	11 (1)	6 (< 1)	7 (1)	
Goiás	48 (1)	13 (1)	13 (1)	13 (1)	9 (1)	
Maranhão	18 (< 1)	4 (< 1)	5 (< 1)	2 (< 1)	7 (1)	
Mato Grosso	19 (< 1)	3 (< 1)	7 (1)	4 (< 1)	5 (< 1)	
Mato Grosso do Sul	16 (< 1)	2 (< 1)	3 (< 1)	4 (1)	7 (1)	
Minas Gerais	224 (4)	51 (4)	57 (4)	66 (5)	50 (4)	
Pará	16 (< 1)	6 (< 1)	0	7 (1)	3 (< 1)	
Paraíba	27 (1)	9 (1)	5 (< 1)	7 (1)	6 (< 1)	
Paraná	107 (2)	27 (2)	19 (1)	30 (2)	31 (2)	
Pernambuco	276 (5)	67 (5)	64 (5)	65 (5)	80 (6)	
Piauí	14 (< 1)	5 (< 1)	2 (< 1)	3 (< 2)	4 (< 1)	
Rio de Janeiro	336 (6)	95 (7)	84 (6)	77 (6)	80 (6)	
R. Grande do Norte	16 (< 1)	3 (< 1)	4 (< 1)	4 (< 1)	5 (< 1)	
R. Grande do Sul	88 (2)	22 (2)	24 (2)	24 (2)	18 (1)	
Rondônia	18 (< 1)	8 (1)	3 (< 1)	5 (< 1)	2 (< 1)	
Roraima	2 (< 1)	0	1 (< 1)	0	1 (< 1)	
Santa Catarina	86 (2)	19 (1)	20 (2)	22 (2)	25 (2)	
São Paulo	3487 (67)	868 (66)	919 (69)	826 (66)	874 (67)	
Sergipe	29 (1)	8 (1)	7 (1)	5 (< 1)	9 (1)	
Tocantins	6 (< 1)	0	1 (< 1)	2 (< 1)	3 (< 1)	
Prior vaccine hesitancy	180 (16)	187 (16)	156 (14)	173 (15)	696 (15)	0.513

*IQR* Interquartile range

^a^missing for 20 participants

^b^missing for 13 participants

^c^missing for 30 participants

^d^missing for 8 participants

^e^missing for 6 participants

^f^missing for 36 participants

### Intention to receive the vaccine against the emerging bogus disease

Four thousand nine hundred three participants (94%) provided valid answers to the question on the intention to receive the vaccine; 313 participants with missing responses and 17 who didn't want to declare were omitted from this analysis. Overall, 88% of participants reported they intended to receive the vaccine for the emerging bogus disease, 4% declared no intention, and 8% were unsure. Table 3A presents frequencies and percentages of each response, along with 95% CI in the complete sample and according to group allocation. The percentage of participants willing to receive the vaccine was higher among those exposed to version 4 (91%, IC 95% 89–92%) and lower among participants exposed to version 1 (84%, IC 95% 82–86%). We performed chi-squared tests contrasting groups 1 vs. 2 to address the effect of adding the case description and photo to the article focusing on the disease characteristics; groups 3 vs. 4 to address the impact of adding the case description and image to the article focusing on the vaccine characteristics; groups 1 vs. 3 to compare the article version focusing on the disease to that focusing on the vaccine; and groups 2 vs. 4 to compare the version focusing on the disease to that focusing on the vaccine when both had the case description and photo. Differences were statistically significant in the two by two comparisons of group 3 vs. group 4 (*p* = 0.045), group 1 vs. group 3 (*p* = 0.014), and group 2 vs. group 4 (*p* = 0.008). The chi-squared test contrasting the percentage of participants willing to receive the vaccine in groups 1 vs. 2 failed to find statistically significant differences (*p* = 0.071).

**Table 3:** Intention to receive the vaccine and to vaccinate offspring against the emerging bogus disease

A: Intention to receive the vaccine					
	Total	Group 1	Group 2	Group 3	Group 4
	*N* = 4903	*N* = 1246	*N* = 1237	*N* = 1189	*N* = 1231
Yes (%; 95% CI)	4290 (88; 87–88)	1046 (84; 82–86)	1078 (87; 85–89)	1047 (88; 86–90)	1119 (91; 89–92)
No (%; 95% CI)	207 (4; 4–5)	72 (6; 5–7)	54 (4; 3–6)	49 (4; 3–5)	32 (3; 2–4)
Don’t know (%; 95% CI)	406 (8; 8–9)	128 (10; 9–12)	105 (8; 7–10)	93 (8; 6–9)	80 (7; 5–8)
*p* = 0.071 for group 1 vs. group 2 comparison *p* = 0.045 for group 3 vs. group 4 comparison *p* = 0.014 for group 1 vs. group 3 comparison *p* = 0.008 for group 2 vs. group 4 comparison					
B: Intention to vaccinate offspring					
	Total	Group 1	Group 2	Group 3	Group 4
	*N* = 732	*N* = 190	*N* = 190	*N* = 173	*N* = 179
Yes (%; 95% CI)	629 (86; 83–88)	159 (84; 78–89)	155 (82; 75–87)	152 (88; 82–92)	163 (91; 86–95)
No (%)	36 (5; 3–7)	7 (4; 1–7)	13 (7; 4–11)	10 (6; 3–10)	6 (3; 1–7)
Don't know (%)	67 (9; 7–11)	24 (13; 8–18)	22 (12; 7–17)	11 (6; 3–11)	10 (6; 3–10)
*p* = 0.379 for group 1 vs. group 2 comparison *p* = 0.514 for group 3 vs. group 4 comparison *p* = 0.094 for group 1 vs. group 3 comparison *p* = 0.031 for group 2 vs. group 4 comparison					

In the analysis of multiplicative interaction using Mantel–Haenszel's homogeneity tests, we found no statistically significant evidence of effect modification between the focus of the newspaper article (vaccine vs. disease characteristics) and the presence of a case description (interaction *p*-value = 0.829). Similarly, we found no statistically significant evidence of interaction between the presence of a patient description and previous vaccine hesitancy (interaction *p*-value = 0.602).

In the analysis of effect modification between the focus of the article and previous vaccine hesitancy, we found that compared to participants exposed to the newspaper article focused on disease aspects, those exposed to the article focused on vaccine characteristics were more likely to declare an intention to receive the vaccine, with a stronger effect among hesitant compared to non-hesitant participants (interaction *p*-value = 0.022; Table 4). Among previously hesitant participants, 12 individuals would have to read a newspaper article focusing on the vaccine characteristics instead of the newspaper article focusing on disease aspects to result in one additional participant declaring an intention to receive the vaccine; among non-hesitant individuals, this intervention would have to be implemented to 50 individuals to obtain the same effect.

**Table 4:** Focus of communication strategy and effect modification by hesitant attitudes

	Hesitant participants *N* = 696		Non-hesitant participants *N* = 3880		
	Intention to receive the vaccine	Prevalence ratio (95% CI)	Intention to receive the vaccine	Prevalence ratio (95% CI)	Prevalence ratio comparing non-hesitant to hesitant participants within strata of communication strategy
Focus on the disease	226 (62%)	1 (reference)	1773 (91%)	1.48* (1.36–1.61)	1.48 (1.36–1.61)
Focus on the vaccine	232 (71%)	1.15* (1.03–1.27)	1800 (93%)	1.51* (1.39–1.64)	1.32 (1.23–1.42)

Measure of effect modification = 5.25, *p* = 0.022

*Prevalence ratio taking as reference the prevalence of intention to receive the vaccine among hesitant participants assigned to the article focused on disease characteristics

### Intention to vaccinate offspring against the emerging bogus disease

Among the 790 participants who declared themselves to be parents or legal caregivers of a child ≤ 5 years old, 732 (93%) provided valid answers to the question on the intention to vaccinate their offspring. Responses are presented in Table 3B. Overall, 86% declared an intention to give the vaccine to their offspring, 5% reported no intention, and 9% were unsure. As observed in responses concerning the intention to receive the vaccine, the percentage of participants willing to vaccinate their children was highest among those exposed to version 4 of the newspaper article. Differences were statistically significant in the two by two comparisons of group 2 vs. group 4 only. We found no statistically significant evidence of interaction between the focus of the newspaper article, the presence of a patient description, or previous vaccine hesitancy for the offspring vaccination intention.

Other responses in the third section of the questionnaire, concerning knowledge/attitudes regarding vaccines, will be analyzed and presented in a separate manuscript.

## Conclusion

In this electronic survey experiment, we included more than 5,000 Brazilian participants to address if different message framing strategies concerning an emerging bogus disease could affect their intention to take the vaccine and to vaccinate their offspring. We found that exposure to a newspaper article focusing on the vaccine safety/efficacy, including a description and picture of a child affected by the disease, was associated with a small but statistically significant higher intention to receive the vaccine compared to a similar article without the case description. The addition of a case description also increased the reported intention to vaccinate among participants exposed to a newspaper article focusing on the disease characteristics; however, this increase failed to reach statistical significance. Finally, exposure to the article focusing on vaccine safety/efficacy was associated with a significantly higher intention to receive the vaccine compared to the article focusing on the disease characteristics, regardless of the presence of a case description. Interestingly, exposure to the article focusing on vaccine safety/efficacy as opposed to disease aspects had a higher effect among participants with prior hesitant beliefs than among non-hesitant participants. In the analysis of intention to vaccinate their children, participants' intentions were also highest among those exposed to the newspaper article focusing on the vaccine safety/efficacy with the description and picture of a child affected by the disease, with statistically significant difference compared to the group exposed to the newspaper article focusing on the disease with the description and picture of a child affected by the disease.

While barriers to vaccine access – such as cost, availability, and service constraints – are still relevant limiting factors for mass vaccination, noncompliance with vaccination recommendations based on a voluntary and deliberate decision—also known as vaccine hesitancy—has been increasingly acknowledged as a driver of declining vaccine coverage in many countries. These challenges have been further intensified by the COVID-1 pandemic, with the politicization of vaccines [9, 10, 18], the mistrust associated with specific manufacturers and countries of origin for the COVID-19 vaccines, fear of side effects of vaccines that use novel platforms, and concerns due to the fast development pace of COVID-19 vaccines [19–22].

Several authors and public health organizations have highlighted that communication, information, and trust are central to reducing vaccine hesitancy [11–13]. However, few experimental studies with inconsistent results have explored specific strategies to facilitate communication, improve the quality of information accessed by the general population or particular groups, and foster trust in vaccine programs [14, 23–26]. Providing epidemiological and tangible information regarding the vaccine side effects was shown to improve vaccine intentions in a study conducted in the USA and the United Kingdom [27]. Messages emphasizing direct individual benefits improved parents' intent to vaccinate their infants with MMR in a randomized study [28]. Another trial including pregnant women showed that, compared to usual care, web-based vaccine information with social media applications was associated with a small but statistically significant increase in childhood vaccination [29]. A motivational interviewing strategy applied to parents during the postpartum stay at a maternity ward improved infants' vaccine coverage up to 7 months of age [30]. A reduction in hesitant attitudes, as measured by the Parent Attitudes about Childhood Vaccines score, was seen among hesitant parents undergoing an educational intervention in a trial conducted in the USA [31]. Contrastingly, a study addressing a physician-targeted communication intervention failed to improve vaccine hesitancy [32].

Tailored communication strategies, considering the audience's specific concerns, values, and racial and cultural characteristics, are more effective in improving vaccine adherence [33–35]. Of note, some studies suggest that messages intended to promote vaccination may sometimes have a harmful effect, depending on the audience, context, and messenger. In a study published in 2014, Nyhan et al. investigated the effectiveness of four different messages designed to reduce misinformation regarding the MMR vaccine among parents of small children in the USA; while none of the interventions significantly increased parental intent to vaccinate a future child, the study found that, among hesitant parents, a message showing pictures of sick children paradoxically increased the belief in the link between vaccine and autism, while the narrative about a severe measles case increased reported belief in serious vaccine side effects. [15]

A few authors have explored the effect of vaccine communication strategies on attitudes toward COVID-19 vaccines. A study investigating the impact of COVID-19 vaccine promotion posters [36] failed to show any improvement in vaccination intent or hesitant attitudes. In a recent research oversampling Black, Latinx, conservative, and religious USA residents, customized messages failed to reduce COVID-19 vaccine hesitancy; moreover, the study found evidence that a vaccine endorsement from Dr. Antony Fauci reduced the intention to vaccinate among participants with a conservative political position [35]. Notably, the COVID-19 pandemic has revealed an unforeseen scenario of the vaccine debate, with discoveries and controversies broadcasted daily in the media, and with the politicization of vaccines [9, 10].

In our study, exposure to information on vaccine efficacy and safety (science-based messaging) was associated with a higher likelihood of vaccination intention compared to exposure to information on disease severity (fear-based messaging). This effect was even higher among previously hesitant individuals. Our results support prior evidence suggesting that hesitant individuals may be even more unwilling to vaccinate after learning about the severity of the disease prevented by a specific vaccine [15]. While challenging to comprehend, this finding may suggest that informing content that sounds intimidating or frightening (such as disease severity) may unexpectedly impair vaccine uptake among hesitant individuals, who could interpret the information as unfounded threats or ill-intentioned fake information. Conjunctly, these findings suggest that the same vaccine-related message may result in an either increased, neutral, or decreased impact on vaccine uptake, depending on the audience, context, and messenger. This discrepancy reveals a familiar challenge to public health: one size does not fit all. To achieve the expected results, vaccine promotion messages should be delivered by trustworthy sources and use persuasive arguments, which can vary from audience to audience. Therefore, when planning communication strategies, the mere provision of information is not enough, and providers should consider the prior beliefs and attitudes of the spectators as fundamental starting points.

Our study had a few limitations. The study questionnaire and bogus articles were created ad-hoc for this study without formal validation assessment. Differences observed in the intention to take the vaccine following exposure to each communication strategy were small; although some have reached statistical significance, our analyses have not included adjustment for multiple comparisons, entailing cautious interpretation. We cannot rule out that aspects beyond the content/message delivered by each article, including the number of words, influenced participants' reported intentions to receive the vaccine and vaccinate their offspring. Most participants were residents of Sao Paulo State, and most were white, older, and highly educated. Other recruitment strategies might have selected participants with a less biased approach regarding socioeconomic status, resulting in a more representative sample of the Brazilian population. Our sample likely had a low percentage of individuals with prior hesitant attitudes compared to the Brazilian population. In addition, communication strategies focusing on the safety and efficacy of a vaccine may be more persuasive to a highly educated population, for whom epidemiological information may sound less intimidating; we cannot rule out that communication strategies focusing on the disease could have resulted in higher intention to vaccinate had we included a less affluent population. As another limitation, our study recruited patients during the most fervent period of the COVID-19 vaccination in Brazil. Besides the potential influence of COVID-19 vaccine-specific hesitant attitudes on questionnaire responses, some reported attitudes might reflect the specific moment of polarized ideologies and intense dissemination of (mis)information, which could significantly change in other contexts. Despite these limitations, we had a high number of participants from all Brazilian federal units and used random allocation of communication strategies, allowing the assessment of the effect of each component of the communication piece (focus on disease vs. vaccine; presence vs. absence of a case description) in a non-biased way. Furthermore, we explored the effect modification by previous vaccine hesitancy, a factor increasingly regarded in the health communication strategies concerning vaccine hesitancy. Finally, we created the fictitious newspaper articles with the support of professional health journalists and disseminated them using social media tools; these strategies likely improved the overall credibility of the text while also matching the means of news dissemination that are most frequently used today.

Developing effective communication strategies is critical to maintain and expand the public health impact achieved by vaccines. Understanding strategies that are more effective in specific populations will help healthcare providers and stakeholders plan and implement effective interventions to ensure the maximum uptake of vaccines in different people, despite prior ideologies or beliefs.

## Data Availability

Currently, the datasets used and analyzed during the study are available from the corresponding author at reasonable request.
